# Novel Mutation at Cys225 in *GNAO1*‐Associated Developmental and Epileptic Encephalopathies: Clinical, Molecular, and Pharmacological Profiling of Case Studies

**DOI:** 10.1002/mco2.70196

**Published:** 2025-05-07

**Authors:** Yonika A. Larasati, Gonzalo P. Solis, Alexey Koval, Marie‐Céline François‐Heude, Julie Piarroux, Agathe Roubertie, Ruihan Yang, Ying Zhang, Dezhi Cao, Christian M. Korff, Vladimir L. Katanaev

**Affiliations:** ^1^ Translational Research Center in Oncohaematology Department of Cell Physiology and Metabolism Faculty of Medicine University of Geneva Geneva Switzerland; ^2^ Service de Neuropédiatrie, Hôpital Gui de Chauliac Montpellier France; ^3^ Institut des Neurosciences de Montpellier, INSERM U 1298 Montpellier France; ^4^ Department of Neurology Shenzhen Children's Hospital Shenzhen China; ^5^ Medical College Shantou University Shantou China; ^6^ Department of Pediatric Neurology the Affiliated Hospital of Qingdao University Qingdao China; ^7^ Medical College Shenzhen University Shenzhen China; ^8^ Pediatric Neurology Unit University Hospitals of Geneva Geneva Switzerland

**Keywords:** developmental and epileptic encephalopathies, G protein, GNAO1, molecular etiology, neomorphic mutations, rare disease

## Abstract

*GNAO1*‐associated disorders have a large spectrum of neurological symptoms, from early‐onset developmental and epileptic encephalopathies (DEE) to late‐onset movement disorders. First reported in 2013 and now identified in around 400 cases worldwide, this disease is caused by dominant, mostly de novo missense mutations in *GNAO1*, the gene encoding the major neuronal G protein Gαo. Being the immediate transducer of a number of neuronal G protein‐coupled receptors, Gαo plays crucial functions in brain development and physiology. Here, we discover a novel mutation site in *GNAO1*, Cys225 mutated to Tyr or Arg in pediatric individuals from France and China (p.(Cys225Tyr) and p.(Cys225Arg), respectively), leading to severe early‐onset DEE. Molecular investigations characterize the novel pathogenic variants as deficient in the interactions with guanine nucleotides and physiological cellular partners of Gαo, with reduced stability and plasma membrane localization and a strong neomorphic interaction with the chaperone Ric8A. Salts of zinc, emerging as a promising targeted therapy for *GNAO1*‐associated disorders, impose a previously unseen effect on the mutant Gαo, accelerating the loss of its ability to interact with guanine nucleotides. Our study, combining clinical, cellular, molecular, and modeling approaches, describes deep insights into molecular etiology and treatment perspectives of the novel form of *GNAO1*‐associated disorders.

## Graphical abstract

The major neuronal G protein Gαo (structural model on the left) is mutated in position Cys225 to Tyr or Arg in a severe form of pediatric encephalopathy. These pathogenic variants produce the protein deficient in physiological cellular interactions, cellular stability, and plasma membrane localization.

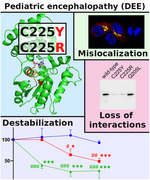

## 1 Introduction

A variety of neurological conditions known as developmental and epileptic encephalopathies (DEEs) affect infants and children with symptoms that include early‐onset refractory epilepsy, developmental and intellectual delay/impairment, and movement disorder. Next‐generation sequencing methods, principally whole‐exome sequencing, identified around 100 genes causative to DEEs so far [1]. *GNAO1*, which codes for the 354 amino acid‐long guanine nucleotide‐binding protein subunit alpha‐o (Gαo), is one of them. Gαo is the major neuronal G protein [2, 3], responsible for signal transduction from numerous G protein‐coupled receptors (GPCRs) involved in nervous system development and functioning [4].

Being a G protein, Gαo cycles through the inactive GDP‐bound and the active GTP‐bound states, whereas the activating GDP→GTP transition occurs by the uptake of GTP that replaces GDP, and the inactivating GTP→GDP transition occurs through the intrinsic GTPase activity of the protein. The rates of these reactions are further controlled by a variety of regulator proteins such as GEFs (guanine nucleotide exchange factors such as GPCRs), GAPs (GTPase‐activating proteins, many of which belong to the class of Regulator of G protein signaling, RGS), and GDIs (guanine nucleotide dissociation inhibitor proteins, most critical of which in the case of heterotrimeric G proteins are the Gβγ subunits that can form together with Gα, in its GDP‐bound state, the heterotrimeric G protein). As can be deduced from their names, GEFs activate G proteins, GAPs deactivate them, and GDIs oppose the activation [5, 6].

Two disorder subtypes are linked with heterozygous dominant *GNAO1* mutations in OMIM: developmental and epileptic encephalopathy 17 (DEE17, OMIM 615473) and neurodevelopmental disorder with involuntary movements (NEDIM, OMIM 617493). Both forms are early‐onset and are accompanied by a strong developmental and intellectual delay [7]. In addition, late‐onset and dystonia‐dominated forms of the *GNAO1*‐associated disorder, with mild developmental and intellectual manifestations, have been found to originate from loss‐of‐function/haploinsufficiency variants [8]. However, more frequent among the ca. 150 distinct variants seen in ca. 400 affected individuals are de novo missense *GNAO1* variants [9]. Attempts to categorize them into loss‐of‐function (LOF) versus gain‐of‐function (GOF) versus dominant‐negative (DN) and to correlate those with clinical manifestations have been performed [10, 11]. However, it has now become clear that on top of a few DN mutations that clinically lead to mild disease phenotypes not so distinct from those due to haploinsufficiency [12, 13, 14], the majority of pathogenic variants are neomorphic in their genetic nature [15, 16, 17].

Although rather ignored in medical genetics, neomorphic mutations have been recognized in classical genetics, from the works of the Nobel laureate Hermann Muller, as one of the five major mutation types (in addition to LOF, partial LOF, GOF, and DN). According to Muller, a “*neomorph represents a change in the nature of the gene … giving an effect not produced, or at least not produced to an appreciable extent, by the original normal gene*” [18]. We proposed the neomorphic nature of severe pathogenic *GNAO1* variants given the confusion in the field, as in different biochemical, cellular, or organism‐level analyses, the same mutations were found to possess features that could be interpreted either as LOF, or GOF, or DN [15, 16]. While searching for the molecular determinant of the neomorphic activities of pathogenic Gαo, we took into consideration the following observations: (1) several Gαo mutants are biochemically inactive after isolation [17, 19]; (2) most mutants have decreased cellular expression levels [10, 13, 14, 17, 20, 21]; (3) despite constitutive GTP loading [13–15, 17, 20, 22, 23], the mutants uniformly fail to interact with RGS proteins [13–15, 17, 19, 22, 24, 25] that normally recognize the GTP‐bound state of Gα‐subunits; (4) pathogenic Gαo variants demonstrate increased flexibility as seen in molecular dynamics simulations [15, 20].

All these features hinted at a potential folding problem, which prompted us to investigate the Ric8A protein, initially described as an activator [26], and then also—as the chaperone for Gα‐subunits of the Gαi/o, Gαq, and Gα12/13 classes [27]. Ablation of Ric8A strongly suppressed levels of their client Gα‐subunits [27]. We have found that *GNAO1* encephalopathy mutants dramatically upscale their cellular interactions with Ric8A—by far above those for Gαo wild‐type (wt) [17]. Further, the mutants re‐localize Ric8A from the cytoplasm to the Golgi apparatus, one of the two intracellular “stations” of Gαo [28, 29]. These findings highlight the genetic nature of the clinically severe *GNAO1* variants as neomorphic (and not LOF or GOF, as previously debated) [15, 16, 17], and identify Ric8A as a “transducer” of the neomorphic activities of pathogenic Gαo. We have also shown that the sequestration of Ric8A by the neomorphic variants of Gαo—which is the major Gα‐subunit in the brain [2, 3]—decreases the chaperone availability to the other Gα‐subunits, thus leading to drops in their expression and signaling levels [17]. This uncovers the totally unanticipated mechanism of disease dominance in *GNAO1* encephalopathy: pathogenic Gαo variants are not only deficient, to different extents, in Gαo‐mediated signal transduction but also infringe the whole Gα‐ and GPCR‐signaling networks in neuronal cells.

As *GNAO1*‐related disorders represent a relatively new condition, with the first individuals harboring *GNAO1* mutations described 12 years ago [30], more cases and more pathogenic *GNAO1* variants become regularly uncovered, and attempts to find the genotype‐phenotype correlation are ongoing [13, 21, 31]. The current work presents two cases from France and China, carrying novel *GNAO1* variants c.674G>A and c.673T>C, affecting the same amino acid Cys225 and producing the variants p.(Cys225Tyr) and p.(Cys225Arg) (C225Y and C225R later on in the article), respectively. These mutations result in severe disease manifestations with seizures, dystonia, and developmental delay. The substitutions of the amino acid 225 cause abnormal intracellular localization and reduced stability of the mutant proteins, accompanied by a quick loss of their GTP‐binding capacity in vitro. The Gαo variants are further deficient in their binding to Gβγ and RGS19—the exemplary binding partners probing the GDP‐ and the GTP‐bound conformations of Gαo, respectively. However, as with many other pathogenic variants [17], C225Y/R display enhanced binding to and relocalization of Ric8A, confirming the neomorphic nature of these novel mutations. Zn^2+^ ions have beneficial effects on the aberrant behavior of several *GNAO1* mutations [15] and are currently in clinical applications [32], leading to a complete loss of the GTP‐binding capacity in the mutants.

The results of this study mark further progress in the clinical and molecular understanding of severe DEEs. Our molecular characterization, coupled with the clinical case descriptions, unravels the molecular etiology of severe pediatric encephalopathies associated with *GNAO1* mutations and offers invaluable knowledge required to tackle this disease in a personalized manner.

## 2 Results

### 2.1 Two Affected Individuals with Mutations at Position Cys225 of GNAO1 Present a Severe Form of DEE

The first individual is a French girl born at term after an uncomplicated gestation. On the first day postpartum, she exhibited chronic seizures affecting all extremities. Managing the initial seizures proved challenging, with a reported episode of status epilepticus within the first few weeks. Complete seizure remission was achieved after subsequent medication adjustments. The individual remained seizure‐free for several months while taking carbamazepine (20 mg/kg/day) and levetiracetam (20 mg/kg/day) (Table 1).

**TABLE 1:** Clinical description of the two clinical cases.

	*GNAO1* variant	Epilepsy Age of onset	Seizure type	Status epilepticus episodes	Most efficacious treatment combination	Movement disorder Age of onset	Abnormal movement type	Most efficacious treatment combination	Development and neurological examination	Brain MRI	EEG Summary of most significant findings
Patient 1	c.674G>A, NM_020988, (p.Cys225Tyr)	Day 1	Bilateral limb clonia	Yes (first weeks of life)	Seizure freedom under: Carbamazepine (20 mg/kg/day) + Levetiracetam (20 mg/kg/day)	1 year	Paroxysmal trunk and limb dystonia	Partial relief under: Cannabidiol (12 mg/kg/day) + Clobazam (0.4 mg/kg/day) + Trihexyphenidyl chlorhydrate (0.2 mg/kg/day)	At 5 years and 4 months: Severe developmental impairment Microcephaly Weakness Axial hypotonia Limb spasticity Transient visual engagement	Normal	Normal or moderate diffuse slowing, no spikes
Patient 2	c.673T>C, NM_020988, (p.Cys225Arg)	Day 6	Dysautonomic signs (facial cyanosis) + limb hypertonia	No	Seizure freedom under: Valproic acid (29 mg/kg/day) + Clobazam (1.2 mg/kg/day) + Topiramate (4 mg/kg/day)	2 months	mild paroxysmal limb hypertonia	N/A	Severe developmental impairment Axial hypotonia	Normal	Multifocal spikes, slow background

At the age of 1 year, the individual presented with recurrent paroxysmal dystonic episodes that affected the body axis and all four limbs and were characterized by sudden, intense trunk hypertonia and limb extension, lasting from several seconds to ten minutes. Topiramate was ineffective, and the symptoms were partially relieved by a combination of cannabidiol (12 mg/kg/day), clobazam (0.4 mg/kg/day), and trihexyphenidyl chlorhydrate (0.2 mg/kg/day). Buccal midazolam could alleviate episodes when administrated soon after the onset of a crisis.

The developmental assessment at 5 years and 4 months revealed competencies comparable to those of a 6‐month‐old infant, including the ability to babble and smile, but lacking independent sitting; mild dysphasia was also present.

Genetic investigations (sequencing of a panel of genes involved in neurodevelopmental diseases, followed by Sanger confirmation in the proband and both her parents) revealed a de novo, heterozygous, missense variant on exon 6 of *GNAO1* (c.674G>A, NM_020988, p.(Cys225Tyr), Hg19chr16:56370723G>A). Following the guidelines of the American College of Medical Genetics and Genomics (ACMG) [33], this variant (C225Y further in the text) was interpreted as likely pathogenic and causative of the clinical presentation.

The second affected individual is a Chinese boy who was delivered via cesarean section due to placental abruption, born to nonconsanguineous parents. On day 3, neonatal jaundice was diagnosed and treated with blue light phototherapy. On day 6, the seizure activity began, which was characterized by facial cyanosis and limb rigidity (Table 1).

Phenobarbital (5 mg/kg/day) was initially used for seizure management but was later replaced with levetiracetam (45 mg/kg/day) and topiramate (7.5 mg/kg/day) due to inefficacy. At 7 weeks, the seizure semiology progressed to electroclinical epileptic spasms. Treatment with adrenocorticotropic hormone (1.2 U/kg/day) was intermittent due to complications; however, a regimen of prednisone (2 mg/kg/day) and valproic acid (18 mg/kg/day) led to improvement. The current treatment plan consists of valproic acid (29 mg/kg/day), clobazam (1.2 mg/kg/day), and topiramate (4 mg/kg/day), which have effectively prevented seizure recurrence.

**FIGURE 1:**
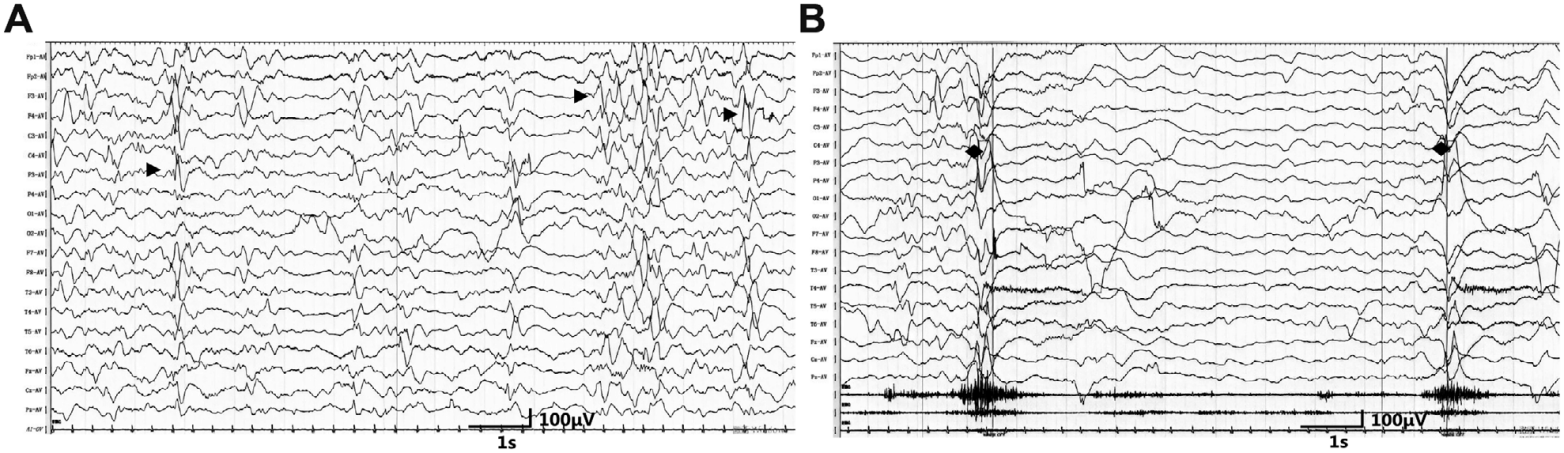
EEG recordings of the patient carrying the C225R mutation. EEG recording of patient 2 performed at 2 months and 22 days of age. (A) interictal tracing, awake recording. Black arrowheads show multifocal spikes. (B) Rhombi show irregular slow waves followed by generalized attenuation and superimposed low amplitude fast waves correlated with the patient's epileptic spasms (see Video in the Supporting Information). Sensitivity: 10 µV/mm, HF: 70 Hz, TC: 0.1 s.

The individual's physical development is delayed. At 2 years and 4 months, he is 90 cm tall and weighs 12.5 kg, paralleling the developmental stage of a 1‐year‐old infant. Severe developmental delay and mild paroxysmal hypertonia affecting both arms and both legs are present. EEG recordings are shown in Figure 1, and an accompanying video file is provided (Video S1).

Trio‐based whole exome sequencing (WES) identified a heterozygous de novo variant in exon 6 of the *GNAO1* gene (NM_020988), specifically the c.673T>C (p.Cys225Arg) variant (C225R further in the text). The variant was confirmed using Sanger sequencing and interpreted as likely pathogenic by the ACMG guidelines [33].

### 2.2 C225Y/R Variants Are Functionally Unstable with Accelerated Destabilization by Zn^2+^

**FIGURE 2:**
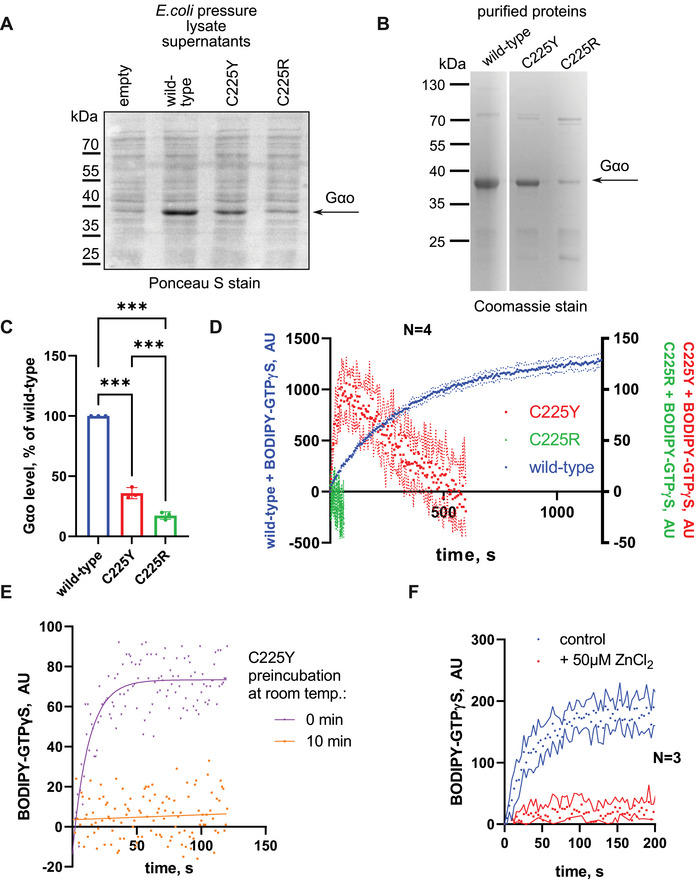
Effects of C225R and C225Y mutations on the GTPase cycle and stability of Gαo. (A–C) Reduced expression levels of C225Y/R in *E. coli*. Ponceau S staining of supernatants from bacterial lysates transferred to nitrocellulose membranes shows that C225Y and C225R mutants accumulate to much lower levels in the bacterial cytoplasm (A, quantification in C), resulting in reduced yields of the purified mutant proteins, especially C225R (B). (D) Fluorescence intensity curves monitoring BODIPY‐GTPγS binding to Gαo variants. C225R shows no detectable nucleotide binding; C225Y shows a decay after the initial peak. Note different Y‐scales for Gαo wt and the pathogenic variants. (E) Comparison of BODIPY‐GTPγS binding to C225Y after preincubation for 10 min at RT or directly from ice storage. (F) The presence of Zn^2+^ further accelerates the loss of the nucleotide binding activity of C225Y. Panels C, D, and F show the results of *N* = 3 independent experiments; error bars or area represent SD. Panel C data were analyzed by one‐way ANOVA with multiple comparisons, significance is shown as ***p < 0.001.

To gain insights into the biochemical properties of the novel pathogenic variants, we bacterially expressed and purified the two mutant forms of Gαo, along with the wild‐type (wt) variant. At the level of this recombinant production, we noticed reduced yields of the mutants, particularly strong for C225R (Figure 2A–C). Monitoring GTP uptake with the BODIPY‐GTPγS assay, we observed no nucleotide binding by C225R and only a poor binding by C225Y (Figure 2D), the latter demonstrating low initial binding followed by a decay. As BODIPY‐GTPγS is nonhydrolyzable, this decay in fluorescence likely reflects instability of C225Y to hold the nucleotide at room temperature (RT) after its initial purification on ice. Indeed, preincubation C225Y at RT for 10 min (the decay time in Figure 2D) completely eliminated the capacity of this variant to bind BODIPY‐GTPγS (Figure 2E). Interestingly, ZnCl_2_ acted on the ice‐cold C225Y similarly to the RT preincubation, completely abrogating its capacity to transiently bind the GTP analog (Figure 2F). We conclude that the two missense mutations at position C225 destabilize the protein, the C225R variant being more severe than C225Y, with zinc treatment further aggravating this destabilization.

### 2.3 C225Y/R Variants Show Reduced Intracellular Stability and Key Cellular Interactions

**FIGURE 3:**
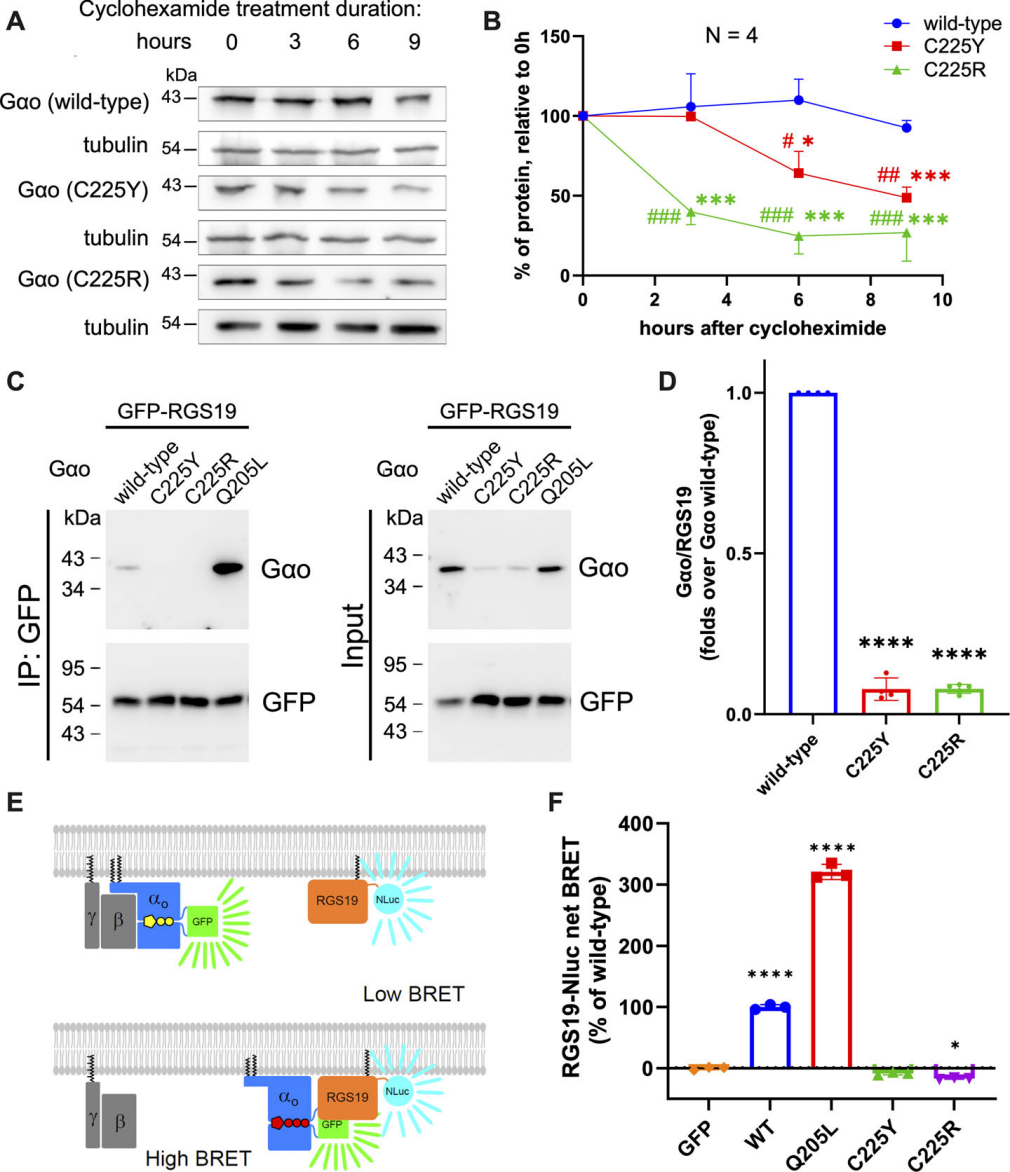
Gαo mutants are intracellularly unstable and lose interaction with RGS19. (A, B) HEK293T cells were transfected with plasmids encoding Gαo wt, C225Y, or C225R and treated with cycloheximide for the indicated time, followed by assessment of the protein levels by Western blotting (A). Quantification of the band intensities (relative to tubulin) plotted as a function of time (B). (C) Co‐IP followed by Western blot analysis of the complex between Gαo variants (including constitutively activated Q205L) and RGS19. Note the lower expression of C225Y/R in the input panel. (D) Quantification of the Gαo‐RGS19 co‐IP experiments. (E, F) BRET‐based assay to monitor interactions between Gαo and RGS19. Statistical evaluation: (B) two‐way ANOVA with multiple comparisons, results are shown as *p < 0.05, **p < 0.01, ***p < 0.001 for comparison with 0 h point and as ^#^ *p* < 0.05, ^##^ *p* < 0.01, and ^###^ *p* < 0.001 for comparison with wt points; data from *N* = 4 independent experiments; (D) one‐way ANOVA followed by multiple comparisons with wt, significance is shown as ****p < 0.0001, data are from *N* = 4–5 independent experiments; (F) one‐way ANOVA followed by multiple comparisons with control (pcDNA transfection), significance is shown as ****p < 0.0001 and *p < 0.05, data are from *N* = 3 independent experiments.

We next analyzed the intracellular stability of the C225Y/R variants. HEK293T cells expressing Gαo wt or C225Y/R were treated with cycloheximide to inhibit de novo protein biosynthesis in order to monitor the kinetics of the eventual decrease in the protein levels, reflecting their intracellular stability. Our results show that the C225Y and especially C225R variants have significantly faster degradation rates compared with wt Gαo, with the mutants’ half‐lives of ca. 9 and 2 h, respectively (Figure 3A,B). This faster degradation is reflected in the overall lower expression levels achieved by the two pathogenic variants (Figure 3C, panel “input”).

We addressed the intracellular functionality of the mutants by analyzing their interactions with two key partners in the GPCR cycle: RGS19 and Gβγ, binding respectively to the GTP‐ and the GDP‐loaded states of Gαo. Interaction with RGS19 was investigated by two different methods: co‐immunoprecipitation (co‐IP) followed by Western blot analysis (Figure 3C,D) and the bioluminescence resonance energy transfer (BRET) assay (Figure 3E,F). The co‐IP analysis revealed that C225Y/R strongly loses the interactions with RGS19. The complementary BRET assay further confirms this conclusion (Figure 3C–F), whereas the constitutively active (not pathogenically seen) Q205L mutant shows strongly increased interactions. We note that the expression level differences between wt and mutant Gαo (Figure 3C, input) complicate these and subsequent analyses despite normalizations performed in the quantification.

**FIGURE 4:**
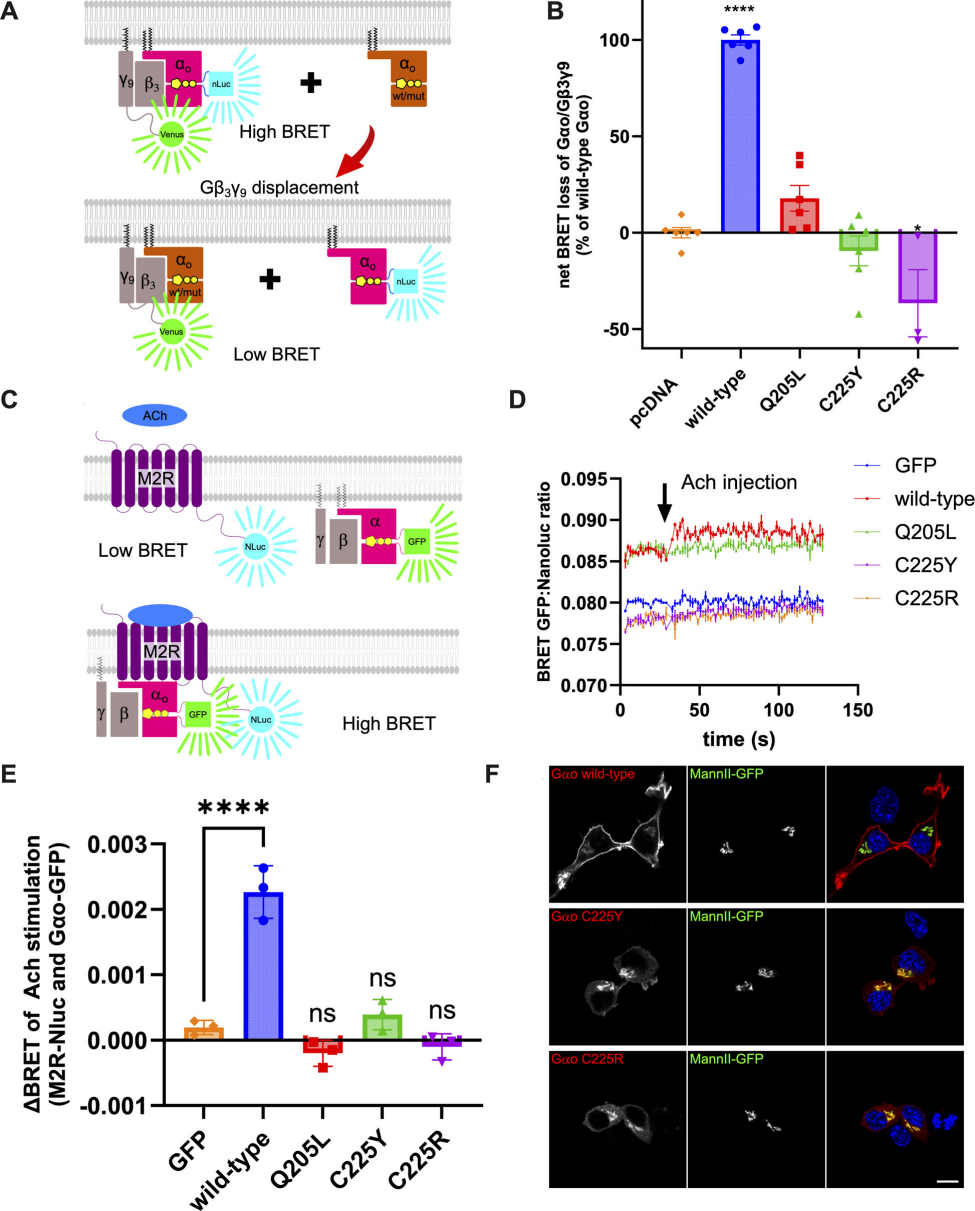
Intracellular localization and interactions with Gβγ and M2R of C225Y/R. (A, B) BRET assay examining interactions between Gαo (NanoLuc‐tagged) and Gβγ (Venus‐tagged γ9 subunit), measuring the capacity of untagged Gαo variants to displace the tagged Gαo from the complex with Gβγ, measured as decreased net BRET signal. C225Y/R and the negative control Q205L are inactive. (C–E) Scheme (C), real‐time kinetics (D), and quantification (E) of interactions between Gαo and M2R GPCR in a BRET assay. (F) Microscopy shows a weak diffuse cytoplasmic pattern of C225Y/R, in contrast to strong plasma membrane localization of wt Gαo. Golgi localization (stained with the MannII‐GFP marker) is similar for all three variants. Statistical evaluation: one‐way ANOVA followed by multiple comparisons with control conditions (pcDNA transfection for (B) and GFP‐encoding vector for (E)). Significance is shown as ****p < 0.0001 and *p < 0.05; data are from *N* = 6 (panel B) or *N* = 3 (panel E) independent experiments. Scale bar: 10µm.

Next, we analyzed the capacity of the mutants to interact with Gβγ. We have previously demonstrated the identical findings emerging from the co‐IP and BRET‐based assays to assess the interactions of Gβγ with pathogenic Gαo variants [12] (Figure 4A). We find that in the BRET assay, the constitutively GTP‐bound Q205L mutant has a vastly reduced capacity to displace the tagged wt Gαo. Both C225Y and C225R are also completely incapable of displacing the tagged wt Gαo from the complex with Gβγ (Figure 4B). Curiously, co‐expression of C225R even results in an increased BRET signal: C225R increases the levels of wt Gαo complexed with Gβγ. This indicates that the mutant forces the wt protein out of certain interactions, making it more abundant for the formation of more heterotrimers.

Next, real‐time kinetics BRET assay was employed to monitor the interaction between Gαo variants and the M2 muscarinic receptor (M2R) as a representative GPCR. Following M2R stimulation by its agonist acetylcholine (Ach), an increase in the BRET output is seen for Gαo wt, presumably due to increased recruitment of Gαo to the GPCR, which serves as an efficient reporter of the functional GPCR‐G protein coupling [15] (Figure 4C). This interaction peaks within approximately 1 s (Figure 4D). In contrast, the two pathogenic variants produced the BRET traces indistinguishable from the negative control, indicating a dysfunctional interaction with the GPCR. Quantification of this rapid increase in BRET signal, expressed as ΔBRET between pre‐ and poststimulation levels, confirms the dysfunctionality of the mutants, identical to the constitutively active Q205L protein used as a control unable to be activated by GPCRs (Figure 4E).

Altogether, we conclude that pathogenic C225Y/R variants lose interactions with the partners recognizing either GTP‐ or GDP‐bound forms of Gαo, suggesting that they are destabilized to the extent of lacking any nucleotide. In this regard, and also due to the loss of the ability of the mutants to form heterotrimeric Gαβγ complexes, it is not surprising that the mutants are unable to engage GPCRs.

### 2.4 C225Y and C225R Mutants Lose Plasma Membrane Localization

We further examined the subcellular localization of Gαo variants (nontagged) by immunofluorescence and confocal microscopy, revealing a marked change in the localization pattern in the mouse neuroblastoma N2a cell line. In contrast to wt protein that shows the dual plasma membrane (PM) and Golgi localization [28, 29], the mutants retain their localization in the Golgi network but lose the PM signal, which is replaced by a diffuse cytoplasmic distribution (Figure 4F).

Interestingly, unlike the C225Y/R mutants, the constitutively active Q205L retains the PM localization [28, 29]. Although Q205L is unable to couple to Gβγ (Figure 4B) and to respond to the Ach stimulation of M2R (Figure 4D,E), the basal signal of Q205L in the BRET assay with M2R is high and equal to wt Gαo (Figure 4D), indicative of the co‐compartmentalization of M2R and Gαo (wt or Q205L) at the PM [34]. In contrast, the basal BRET signal for C225Y and C225R is identical to that obtained for cytoplasmic GFP, agreeing with the results of microscopy‐based observations that the two mutants lose their PM localization.

### 2.5 The C225Y/R Variants Show Neomorphic Interaction with and Mislocalization of Ric8A

**FIGURE 5:**
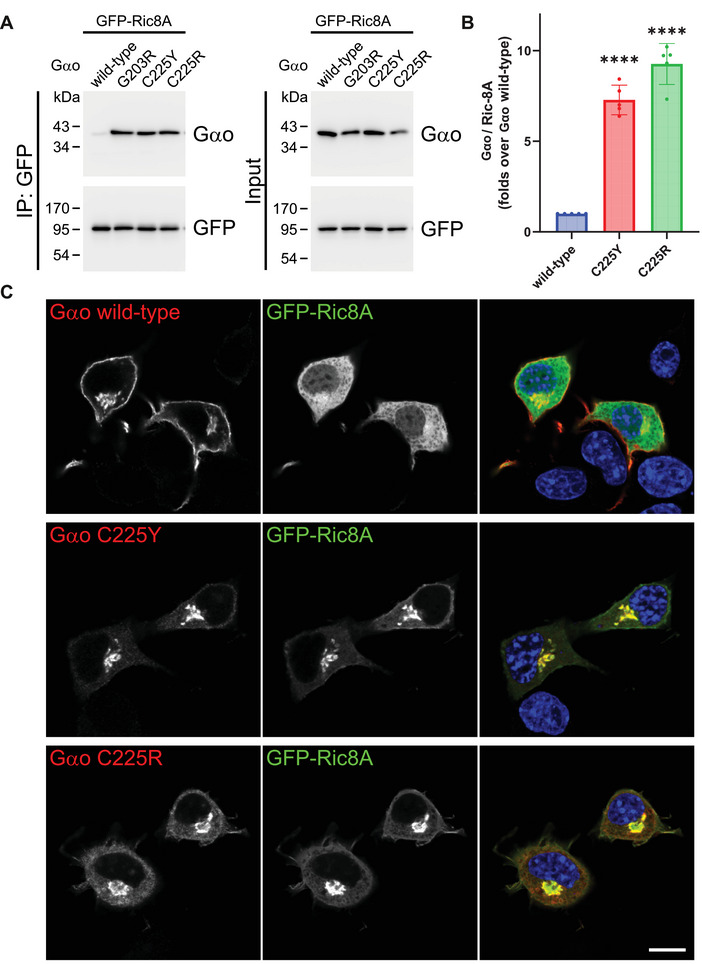
C225Y/R bind and relocalize Ric8A. (A, B) Co‐IP followed by Western blot examining the interactions between Gαo and Ric8A. C225Y/R stronger bind to Ric8A, similar to the highly pathogenic G203R used as a positive control. (C) Confocal microscopy images of subcellular localization of Ric8A in N2a cells co‐expressing GFP‐tagged Ric8A and untagged Gαo variants. Ric8A is cytoplasmic in cells with wt Gαo and colocalizes to Golgi with the mutants. Scale bar: 10 µm. Statistical evaluation: one‐way ANOVA followed by multiple comparisons; significance is shown as ****p < 0.0001. Data are from *N* = 5 independent experiments.

Our findings so far indicate that C225Y and C225R may reside in cells in a nucleotide‐free state. Ric8A is a chaperone of Gαo and other Gα‐subunits that interacts with Gα in its nucleotide‐free state [26, 27], and we have previously shown that most pathogenic Gαo variants massively gain constitutive interaction with Ric8A [17]. We thus tested whether C225Y/R interacted with Ric8A, performing co‐IP experiments from N2a cells transfected with untagged wt or mutant Gαo and a GFP‐tagged Ric8A. We found that both variants massively bind Ric8A, about an order of magnitude over wt (Figure 5A,B), comparably to the highly pathogenic G203R variant used as a positive control [17].

Next, we examined the subcellular localization of Ric8A in the presence of Gαo variants, observing Ric8A mainly distributed in the cytoplasm in cells co‐expressing wt Gαo, consistent with our previous findings [17]. In contrast, Ric8A is mislocalized, to similar extents, to the Golgi in cells expressing the C225Y/R (Figure 5C). These features of the C225Y/R variants—massive binding to Ric8A and its relocalization to Golgi—make them stand together with the most clinically severe *GNAO1* mutations we analyzed previously [17].

### 2.6 In Silico Modeling Suggests that the C225Y/R Mutants Lose the Ability to Bind GTP Through Disturbance of the P‐loop

**FIGURE 6:**
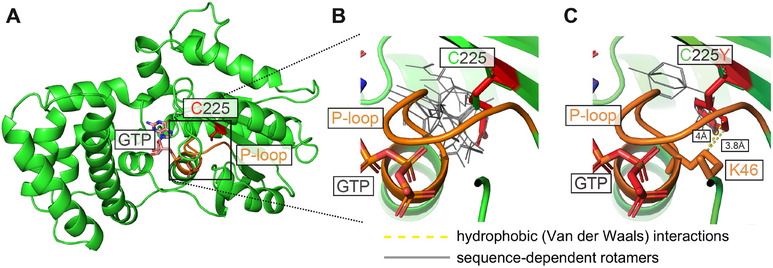
In silico analysis of C225Y/R. (A) Overview of wt Gαo structure, highlighting the position of C225 in the overall fold and its proximity to P‐loop involved in binding GTP’ phosphate groups. (B) Close‐up of C225R, showing the bulky side chain of Arg (red), significantly increased over the relatively short Cys (green), but fully accommodated and without new interactions. R225 gains 24 potential rotamers (gray wire) at this position, directed toward the P‐loop. (C) Close‐up of C225Y: The Tyr substitution gains 4 rotamers (gray wire) that support hydrophobic interaction with the aliphatic part of the K46 side chain.

To gain insight into the molecular mechanism behind the observed defects of the two pathogenic variants, in silico structural modeling was performed (Figure 6). The original amino acid Cys225, although not displaying any specific prior interactions that might be disrupted in the mutant proteins, is localized in close proximity to the so‐called P‐loop—the phosphate‐binding critical structural motif found in GTPases and ATPases (Figure 6A) [35]. In heterotrimeric G proteins, the P‐loop (GXXXXGK(S/T)) interacts with the phosphate groups of the guanine nucleotides, stabilizing the nucleotide binding. Modeling suggests that the two mutations, despite the introduction of the bulkier (as compared with Cys225) side chains of Arg225 and Tyr225, do not significantly change the overall energy‐minimized protein conformation or the conformation of the P‐loop, as there is enough space to accommodate them (Figure 6B,C). However, impacts on the nucleotide binding can be deduced from the structural analysis. Specifically, for the highly flexible side chain of Arg225, 24 potential rotamers (different side chain positions) emerge in the energy‐minimized states of the model as accommodatable by the sequence context in the vicinity of the mutation site (Figure 6B). As rotamers represent energetically favorable side chain conformations, they indicate the likely scope of the residue's motility, which in the case of Arg225 goes toward the nearby P‐loop. We predict that this conformational flexibility of Arg225 sterically clashes with the P‐loop. For Tyr225, modeling predicts only 4 permitted rotamers but shows that through their hydrophobic part of the side change, they establish a novel van der Waals interaction with Lys46 of the P‐loop (Figure 6C), which likely impinges on the positioning of the loop. Taken together, these results suggest that while the mutations do not affect the protein's backbone, they most likely modulate P‐loop dynamics through indirect steric effects and potential hydrophobic contacts.

## 3 Discussion

This study investigates the effects of two novel pathogenic variants, C225Y and C225R, in the major neuronal G protein Gαo encoded by the *GNAO1* gene. These mutations have been identified in pediatric individuals from France and China, and cause a severe neurodevelopmental disorder characterized by epilepsy, movement disorder, and developmental delay. The study finds that both mutations impair the ability to handle guanine nucleotides and stability of Gαo, as well as its interactions with key partners such as Gβγ, RGS19, and GPCRs. In addition, C225Y/R lose their plasma membrane (PM) localization. We have earlier shown that among many pathogenic *GNAO1* variants, those losing their PM localization invariably lead to clinical manifestations that include epilepsy, while those that clinically lead to the motor dysfunction‐only phenotypes retain their PM localization [13, 17]. The genotype–phenotype correlation of the new C225Y/R variants obeys this rule.

We further find evidence that mutations in the position C225 destabilize Gαo, with the C225R variant being more severely affected than C225Y. Indeed, these pathogenic variants are expressed at reduced levels in HEK293T cells as well as in bacteria; the stability of these variants in HEK293T cells is strongly reduced; after isolation, C225R is unable to bind GTP, while C225Y rapidly loses this ability at room temperature. These features group C225Y/R together with some other variants, namely G40R, G45E, Q52P, Q52R, D174G, N270H, F275S, and I279N we studied earlier [17, 19].

Notably, the accelerated degradation of the C225Y/R proteins still takes hours (half‐life of ∼9 h for C225Y and ∼2 h for C225R), making this variant more stable, than e.g., the P170R mutant, which has a very poor intracellular stability with the half‐life of less than 1 h [25]. Further, the intracellular degradation of C225Y does not correspond to the fast loss of the GTP‐binding capacity observed in our assays with the purified proteins, where the destabilization takes minutes. These findings may indicate that the degradation rate observed in vivo is distinct from the loss of the biochemical activity, and thus, the biochemically inert fraction is likely to be stable intracellularly and to represent a major fraction of the protein. We further speculate that this cellular inactive fraction of C225Y/R represents a nucleotide‐free form of the protein. This hypothesis is supported by the observation that the mutants drastically lose the ability to interact with key partner proteins that normally probe either the GDP‐bound state of Gαo (Gβγ) or its GTP‐bound state (RGS19). Structural modeling offers the likely atomistic explanation of this major defect of the C225Y/R variants, whereas the two substitutions destabilize the P‐loop in Gαo—the motif conserved among GTPases and ATPases and required for the proper binding of nucleotides [35]. Interestingly, the same mechanism is likely in place in the case of the pathogenic variants G40E and G45E, which fall within the P‐loop, and Q52P and Q52R that directly follow the P‐loop, all producing similarly GTP‐binding incompetent proteins [17, 19].

The reduced stability of C225Y/R and its possible nucleotide‐free state within cells brought us to investigate the interactions with Ric8A, a protein that acts as a chaperone and regulator of Gα subunits of Gαi/o, Gαq, and Gα12/13 classes [17, 26, 27]. These Gα‐subunits are believed to engage in interaction with Ric8A during translation, whereas Ric8A assists in proper folding of the nascent Gα chain, initially in the nucleotide‐free state, and then permits binding of the ‘first’ GTP by the Gα, the event leading to dissociation of the Gα‐Ric8 complex that sends the mature Gα toward its “adult” life [27, 36, 37]. Indeed, we find that C225Y/R gain massive interaction with and Golgi relocalization of Ric8A. The significance of the neomorphic pool of the Ric8‐Gαo complexes can be inferred from our BRET data on Gαo interactions with Gβγ: the C225R variant not only fails to displace wt Gαo from its complexes with Gβγ but actually increases the quantity of these wt heterotrimers (Figure 4B). This finding suggests that wt Gαo is displaced from the Ric8A complexes by Gαo[C225R], redirecting wt Gαo from the Ric8A‐bound pool to the Gβγ‐bound pool. Interestingly, we find that co‐expression of Ric8A strongly increases the cellular levels of C225Y/R (cf. the “input” panels in Figures 3C and 5A). The dramatically increased association with Ric8A and Golgi mislocalization of this chaperone protein group C225Y/R together with the most clinically severe, neomorphic *GNAO1* mutations [17].

Salts of zinc have emerged as a promising therapeutic approach going to the core of the molecular dysfunction caused by pathogenic *GNAO1* variants [15, 32, 38]. By replacing Mg^2+^ in the active center of Gαo, Zn^2+^ induces structural rearrangements in the mutant (but not wt) protein, restoring the deficient guanine nucleotide handing and cellular interactions [15]. In a *Drosophila* model of the disease, characterized by severe motor dysfunction and reduced life span, dietary zinc supplementation provides a strong rescue in these disease phenotypes [15, 39]. Given the fact that salts of zinc are an approved treatment for a multitude of indications including pediatric disorders [40, 41], given the safety of zinc supplementation in the neonatal mouse model [32], and finally given the excellent safety profile and a significant amelioration of the disease conditions in the first‐in‐human trial in a pediatric individual carrying the G203R *GNAO1* allele [32], clinical trials of zinc application to *GNAO1* cases have been launched in China and Europe (Prospective pilot trial to address the feasibility and safety of treatment with oral zinc in GNAO1 associated disorders (ZINCGNAO1), registered: NCT06412653; EUCT Nr. 2024‐512735‐72‐00). Thus, the natural question emerging from our current study is whether the treatment with zinc could be beneficial to the individuals we describe here, carrying the novel C225Y/R mutations.

Our recent research has revealed that a large list of pathogenic missense *GNAO1* variants can be grouped into three classes by their responsiveness to zinc in a biochemical setting [32]. The first, exemplified by the mutations resulting in relatively mild clinical manifestations such as L23P, C215Y, I344del, and the intronic mutation (NM_020988.3:c.724‐8G>A) leading to the T241_N242insPQ variant with an in‐frame insertion of two amino acids, show complete biochemical insensitivity to zinc, just as wt Gαo is unresponsive to the ion [15, 24, 32]. The second class is represented by the K46R, G203R, R209C, and E246K mutants that are deficient in their GTPase activity, the deficiency reverted by zinc [15, 32]. Finally, the third class of mutations (K46N, H57P, T182I, P170R, R209H, Y231C, E237K, and Y291N) that are also constitutively GTP bound through accelerated GTP uptake and compromised GTP hydrolysis, respond to zinc by the reduction in the affinity to GTP, which leads to their deactivation through accelerated loss of the bound nucleotide [25, 32].

However, another group of pathogenic *GNAO1* variants has so far evaded this classification. Indeed, such mutants as G40R, G45E, Q52P, Q52R, D174G, N270H, F275S, and I279N we studied earlier [13, 17, 19], and now C225Y and C225R, are biochemically ‘dead’ upon purification, and thus technically could not be studied in our biochemical assays in terms of their responsiveness to zinc. Here, we attempted to overcome this hurdle by using the “partially dead” variant C225Y that has the initial GTP‐binding activity, rapidly decaying within 10 min of the experiment. We added ZnCl_2_ to this variant for this initial active period, to find that Zn^2+^ completely abrogates this atavistic activity of the mutant. Two biochemical explanations could be proposed to explain this effect. The first is that Zn^2+^ speeds up the deactivation of Gαo[C225Y], making it incapable of binding GTP. This scenario would place C225Y, and by inference, potentially, other biochemically inactive variants, into a fourth category in terms of their sensitivity to zinc: the mutants whose deactivation is catalyzed by Zn^2+^. The second explanation is that Zn^2+^ dramatically reduces the affinity of the mutant to GTP, thus, we see no binding in our biochemical experiments. In this scenario, C225Y (and potentially the other variants) would join the 3rd category of mutants. More experiments are required to discriminate between these two models regarding the mechanism of zinc action of C225Y and other inactive pathogenic variants. Regardless of this, we can conclude that C225Y and, plausibly, C225R are responsive to zinc, unlike the class I mutants. Noteworthy, the clinical severity of the C225Y/R variants would agree with our prior observations that the zinc responsiveness correlates with the strength of the disease manifestations [32].

Whatever the exact biochemical mechanism of action of zinc on the C225Y/R variants, the clinically relevant question is whether we may expect a therapeutic effect of the zinc treatment in the individuals carrying these pathogenic variants. At this stage, without *C. elegans* [42], Drosophila [15], or mouse [23, 43] models of these exact mutations, we cannot provide an answer. We hope that the outcome of the clinical trials ongoing for individuals with a large panel of *GNAO1* mutations will provide crucial safety and therapeutic clues of this treatment approach, clues that will guide the clinician's decisions regarding the two pediatric cases we describe here.

Overall, our study offers new insights into the genotype‐phenotype correlation of the *GNAO1*‐associated epileptic encephalopathy. The new site—Cys225—we discovered mutated to two different pathogenic amino acids, Tyr and Arg, in affected individuals from Europe and Asia, identifying yet another sensitive region in Gαo. By uncovering the molecular and cellular deficiencies imposed by these mutations, we bring us closer to the hopeful clinical success in treating and preventing this devastating rare disease.

## 4 Materials and Methods

### 4.1 Patients

The study was approved by the Montpellier University Hospital Ethical Committee (MR004: 2019_IRB‐MTP_11‐23) and the Clinical Research Ethics Committee of Shenzhen Children's Hospital (no. 202404702). The parents gave their written informed consent according to the Declaration of Helsinki for the review and publication of the clinical information, including EEG results and the video. The patients were followed by clinicians of Hospital Gui de Chauliac in Montpellier, France (patient 1) and of Shenzhen Children's Hospital in Shenzhen and Affiliated Hospital of Qingdao University, Qingdao, China (patient 2).

### 4.2 Plasmids

The plasmids for nontagged Gαo in pcDNA3.1+, His_6_‐Gαo (N‐terminal His_6_‐tag), Gαo^G92^‐GFP (internal GFP‐tag at Gly92), Go1‐CASE, GFP‐Gβ1, GFP‐Gγ3, M2R‐NLuc, MannII‐GFP, and GFP‐Ric8A were previously reported [6, 17, 19, 28, 44]. For the generation of the EGFP‐RGS19 construct, the RGS19 sequence was cut with BamHI and PspOMI from the His_6_‐RGS19 plasmid [6] and ligated in frame into the site of BglII/PspOMI of pEGFP‐C1 plasmid [28]. For the NLuc‐RGS19 construct, the NLuc sequence was cut with NheI and BsrGI from the pNLuc‐C1 plasmid [28] and ligated in frame into the NheI/BsrGI sites of the pEGFP‐RGS19.

For site‐directed mutagenesis, the forward and reverse primers were as follows (mutation position is in the small font):

C225Y_f GCCATCATTTTCTaTGTCGCGCTCAGCGGCTATGACCAG
C225Y_r TGAGCGCGACAtAGAAAATGATGGCCGTGACGTCCTCG
C225R_f GCCATCATTTTCcGTGTCGCGCTCAGCGGCTATGAC
C225R_r CTGAGCGCGACACgGAAAATGATGGCCGTGACGTCCTC

Using Phusion DNA Polymerase and the protocol provided by the manufacturer, the primers were used for whole plasmid PCR amplification with an annealing temperature of 60°C and an extension time of 7 min. The methylated parental plasmid DNA was digested by DpnI enzyme treatment of the PCR product, which was then immediately transferred to competent *E. coli* Top10 cells. The colonies that developed overnight on LB agar plates with the correct antibiotic were picked and cultured, and the plasmid DNA isolated from them was sequenced to verify the integrity of the insert and the presence of the mutation.

### 4.3 Bacterial Expression

Using conventional techniques and our previously described pET23b‐based *E. coli* expression system [15, 24], we isolated recombinant wt Gαo and its two pathogenic variants. After induction with 0.25 mM isopropyl‐β‐D‐thiogalactopyranoside (Biosynth, EI05931) of the Rosetta(DE3)pLysS strain (Novagen, 70956) at 18°C, the culture was allowed to grow for 14–18 h for protein production. All subsequent steps were performed on ice or at 4°C using prechilled equipment and buffers. Using a pressure lyser (Constant Systems One Shot), harvested and resuspended bacteria were lysed in TBS (20 mM Tris‐HCl, pH7.5 and 150 mM NaCl) supplemented with 1 mM PMSF and 30 mM imidazole. The supernatant was cleared by centrifugation at 15,000×*g* for 15 min and incubated overnight with Ni‐NTA agarose beads on a rotary shaker. The next day, the beads were washed three times with 10 resin volumes of wash buffer (TBS supplemented with 10 mM imidazole), and bound proteins were GDP‐loaded in TBS supplemented with 3% glycerol, 10 mM MgCl_2_, 0.1 mM DTT, and 200 µM GDP. Beads were washed three more times with at least 10 resin volumes of wash buffer before elution with TBS containing 300 mM imidazole. Vivaspin centrifugal concentrators were used to extract imidazole by replacing the buffer with TBS. Protein concentration was determined by the Bradford assay, and purity was determined by SDS‐PAGE followed by Coomassie staining.

### 4.4 Nucleotide Binding

To assess the guanine nucleotide handing by the three Gαo variants, we used the nonhydrolyzable fluorescent GTP analog BODIPY‐GTPγS, which increases the fluorescence yield upon protein binding, allowing the real‐time monitoring of the kinetics of the nucleotide uptake [6, 45]. The fluorescence increase typically corresponds to an exponential process with a plateau, permitting the assessment of the kinetic rate constant *k*_bind_ that is strongly increased for the majority of pathogenic Gαo variants [13, 15, 17]. In contrast, the shape of the curve in the C225Y experiment (the initial rise followed by a drop in fluorescence, Figure 2D) does not permit the assessment of *k*_bind_ as there is no plateau in this experiment.

The BODIPY‐GTPγS assay was performed as described [6, 15, 24]. Briefly, recombinant Gαo was diluted to 1µM in the assay buffer [TBS supplemented with 10 mM MgCl_2_ and 0.5% bovine serum albumin (BSA)]. The mixture was pipetted into black 384‐well plates (Greiner), and BODIPY‐GTPγS (Invitrogen, final concentration 1 µM) was injected into the wells immediately before measurement. Fluorescence measurements were performed at 28°C in a Tecan Infinite M200 PRO plate reader with excitation at 485 nm and emission at 530 nm. For assay using ZnCl_2_, Gαo (1 µM) and ZnCl_2_ (50 µM, Sigma‐Aldrich) were mixed and pipetted into black 384‐well plates before the addition of BODIPY‐GTPγS as described above.

### 4.5 Cell Lines and Culture Conditions

Human HEK293T (ATCC, CRL‐3216) cells were grown in Dulbecco's modified Eagle's medium (DMEM; Thermo Fisher Scientific) supplemented with 10% fetal calf serum, 2 mM L‐glutamine, and 1% penicillin‐streptomycin. Mouse neuroblastoma Neuro‐2a cells (N2a; ATCC, CCL‐131) were maintained in minimum essential medium (MEM; Thermo Fisher Scientific), supplemented as above and 1 mM sodium pyruvate. Cells were kept at 37°C and 5% CO_2_ under humidity. Plasmid transfections were carried out with X‐tremeGENE HP (Roche, XTGHP‐RO) or TransIT‐2020 (Mirus, MIR5400) according to the instructions of the manufacturers. The cell lines are confirmed to be mycoplasma‐free.

### 4.6 Immunoprecipitations

For immunoprecipitation (IP), HEK293T cells were co‐transfected for 24 h with GFP‐tagged constructs (GFP‐Gβ1γ3, GFP‐RGS19, or GFP‐Ric8A) and nontagged Gαo (wt, C225Y, or C225R) to equal plasmid ratios. IPs were performed using a recombinant GST‐tagged Nanobody against GFP as previously described [17, 19]. The pulldown of GFP‐Gβ1Gγ3 (*n* = 2), GFP‐RGS19 (*n* = 4–5) and GFP‐Ric8A (*n* = 5) were analyzed by Western blot using a rabbit polyclonal antibody against GFP (1/2000; PABG1, Proteintech) and a mouse monoclonal against Gαo (1/100; E1, Santa Cruz Biotechnology). The quantification of blots was done using ImageJ‐v1.54f, and images were edited using EvolutionCapt‐v18.11 (Vilber).

### 4.7 Immunofluorescence and Microscopy

The subcellular localization of nontagged Gαo constructs was analyzed in N2a cells by immunofluorescence and microscopy as previously published [12, 17]. Briefly, N2a cells were co‐transfected for 24 h with Gαo constructs and MannII‐GFP (10:1 plasmid ratio) or GFP‐Ric8A (1:2 plasmid ratio). Immunostaining was done using the mouse monoclonal antibody against Gαo (1/50; A2, Santa Cruz Biotechnology) and stained with DAPI (Sigma‐Aldrich) to label nuclei. Cells were recorded in LSM800 Confocal Microscope using the ZEN 2.3 software (Zeiss).

### 4.8 Bioluminescence Resonance Energy Transfer Assay

HEK293T cells (48,000 cells/well) were seeded on 48‐well plates. The Gβ3γ9 displacement assay was performed as previously reported [17], utilizing internal tagging of Gαo by GFP at position Gly92, whereas the Gβ3γ9 heterodimer harbors the Venus tag on the C‐terminus of the Gγ9 subunit [44]. In this setting, co‐expression of untagged Gαo results in a significant loss of the BRET signal by displacing the tagged subunit from the heterotrimeric complex, which is taken as 100% in the assay. The M2R‐based BRET assays, where M2R was tagged by NanoLuc and the Gαo variants were internally tagged by GFP, were also performed as reported [17]. The novel BRET assay monitoring Gαo‐RGS19 interaction is built using internal tagging of Gαo tagged at Gly92 with GFP and NanoLuc‐tagged RGS19.

For the Gβ3γ9 displacement assay, cells were co‐transfected with the Go1‐CASE plasmid and pcDNA3.1 or nontagged Gαo at a 1:3 ratio. For the M2R‐based BRET, cells were co‐transfected with the M2R‐NLuc plasmid and GFP or GFP‐Gαo at a 1:3 ratio. For the RGS19‐based BRET, cells were co‐transfected with the NLuc‐RGS19 plasmid and GFP or GFP‐Gαo at a 1:5 ratio. Twenty‐four hours after transfection, cells were seeded at 12,000 cells/well in the transparent‐bottom black 384‐well plates. After an additional 24 h, the medium was replaced by 10 µL of PBS. Furimazine was injected to 10 µM immediately before measurement, and reading was performed with a Tecan Infinite plate reader using a built‐in NanoBRET filter. BRET signal is determined by calculating the ratio of the light emitted by the GFP‐Gαo over the light emitted by the NLuc. For M2R‐based BRET, Acetylcholine (10 µM; Sigma‐Aldrich) was injected sequentially at the indicated times. The average baseline value (basal BRET ratio) recorded prior to agonist stimulation was subtracted from the experimental BRET signal values to generate ΔBRET.

### 4.9 Determination of Protein Stability with Cycloheximide

HEK293T cells were transfected with pcDNA3.1+ vector encoding untagged Gαo (wt, C225Y, or C225R). At 24 h posttransfection, the cells were treated with cycloheximide (CHX) at a final concentration of 50 µg/mL to stop protein synthesis, followed by incubation at 37°C for the time points indicated in each figure. The cells were then lysed with RIPA buffer (150 mM NaCl, 1% Triton X‐100, 0.5% sodium deoxycholate, 0.1% sodium dodecyl sulfate (SDS), and 50 mM Tris), and after centrifugation, the supernatants were analyzed by Western blotting and quantified.

### 4.10 Structure Modeling and Analysis

Molecular modeling of the mutant protein was performed using the default parameters of the SWISS MODEL server, and the resulting structure was analyzed and presented in PyMol. The molecular model of mutant Gαo was generated in its energy‐minimized state using ProMod3/OpenStructure for homology modeling, with Gαi3 (PDB ID 8gvx), followed by optimization of the rotamers using TreePack and SCWRL4 energy reduction and refined energy minimization using OpenMM and the CHARMM22/CMAP force field. The ligand position was taken from the template if there were no steric conflicts.

### 4.11 Statistical Analysis

Data in this study are represented as mean ± SEM. A statistics analysis was performed using Prism v10.3.1, and the type of analysis is listed in each figure legend.

## Author Contributions

Clinical description of the affected individuals: Marie‐Céline François‐Heude, Julie Piarroux, Agathe Roubertie, Ruihan Yang, Ying Zhang, Dezhi Cao, and Christian M. Korff. Experimental analysis of biochemical, cellular, and structural aspects of the pathogenic variants: Yonika A. Larasati, Gonzalo P. Solis, and Alexey Koval. Design and supervision of the work: Christian M. Korff, Vladimir L. Katanaev. Writing the manuscript: Alexey Koval and Vladimir L. Katanaev. All authors have read and approved the final version of the manuscript.

## Ethics Statement

Written consents were obtained from the patients’ parents to perform this clinical‐molecular investigation and to publish its results, including video. The study was approved by the Montpellier University Hospital Ethical Committee (MR004: 2019_IRB‐MTP_11‐23) and the Clinical Research Ethics Committee of Shenzhen Children's Hospital (no. 202404702).

## Conflicts of Interest

The authors declare no conflicts of interest.

## Supporting information



Supporting Information

## Data Availability

All data related to this study are provided in full in the main text, table, and figures of this article.
